# Supplementary material for: Co-Aggregation of S100A9 with DOPA and Cyclen-Based Compounds Manifested in Amyloid Fibril Thickening without Altering Rates of Self-Assembly
Source: Int J Mol Sci. 2021 Aug 9;22(16):8556. doi: 10.3390/ijms22168556 (PMC8395260; doi:10.3390/ijms22168556)
Supplement: Supplementary file 1 [file ijms-22-08556-s001.zip › Supplementary Materials 2021 0807.pdf]

Supplementary Materials

Co-Aggregation of S100A9 with DOPA and Cyclen-Based Compounds Manifested in Amyloid Fibril Thickening without Altering Rates of Self-Assembly

Lili Arabuli [1,2], Igor A. Iashchishyn [1], Nina V. Romanova [1], Greta Musteikyte [3], Vytautas Smirnovas [3], Himanshu Chaudhary [1,*], Željko M. Svedružić [4,*] and Ludmilla A. Morozova-Roche [1,*]

1 Department of Medical Biochemistry and Biophysics, Umeå University, Umeå, SE-90781, Sweden; l.arabuli@ug.edu.ge, igor.iashchishyn@umu.se; nina.romanova@umu.se; himanshu.chaudhary@umu.se; ludmilla.morozova-roche@umu.se

2 School of Science and Technology, Department of Natural Sciences, University of Georgia, Tbilisi, 0171, Georgia; l.arabuli@ug.edu.ge

3 Institute of Biotechnology, Life Sciences Center, Vilnius University, Vilnius, LT-10257, Lithuania; vytautas.smyrnovas@bti.vu.lt; gm629@cam.ac.uk

4 Department of Biotechnology, University of Rijeka, Rijeka, HR 51000, Croatia; Department of Biotechnology, University of Rijeka, Rijeka, HR 51000, Croatia; zeljko.svedruzic@biotech.uniri.hr

* Correspondence: ludmilla.morozova-roche@umu.se; Tel.: (+46907865283 L.A.M.-R.); zeljko.svedruzic@biotech.uniri.hr; himanshu.chaudhary@umu.se

*

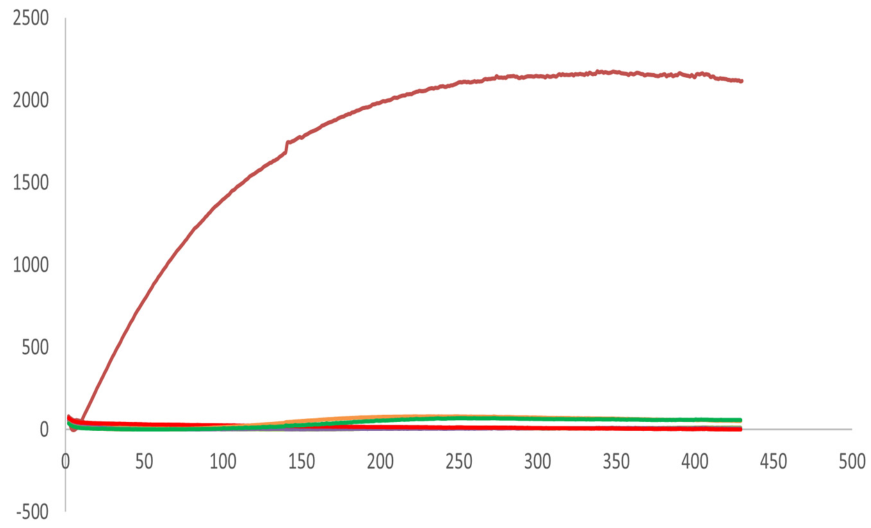

Figure S1. Time dependence of Thioflavin-T fluorescence changes during its incubation with S100A9 and DOPA and Cyclen-based compounds. Thioflavin-T fluorescence in arbitrary units is shown along *y*-axis, time in minutes – along *x*-axis. Increase in Thioflavin-T fluorescence in the presence of 75 µM S100A9 reflects its amyloid self-assembly and shown in brown. By contrast, there were no increase in Thioflavin-T fluorescence upon incubation with 75 µM of corresponding compounds as shown by flat lines close to zero, where time dependences for DOPA-D-H-DOPA us shown by bright green line, for DOPA-H-H-DOPA – by orange, DOPA-D-H – by chartreuse, H-E-Cyclen – by red and DOPA-Cyclen – by purple. These lines are mostly overlap, indicating that there is no amyloid self-assembly of these compounds under the conditions of our experiments. PBS, pH7.4 and 42 ^{o}C.

DOPA-D-H-DOPA	DOPA-H-H-DOPA	DOPA-D-H	H-E-Cyclen	DOPA-Cyclen
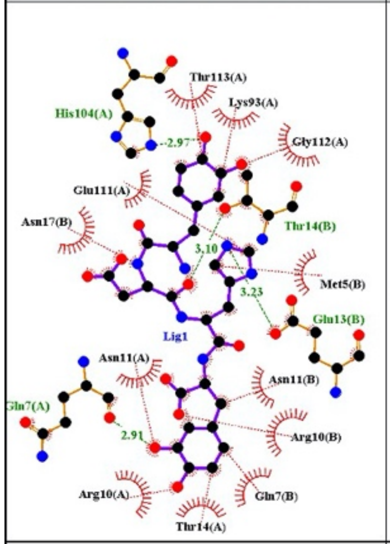	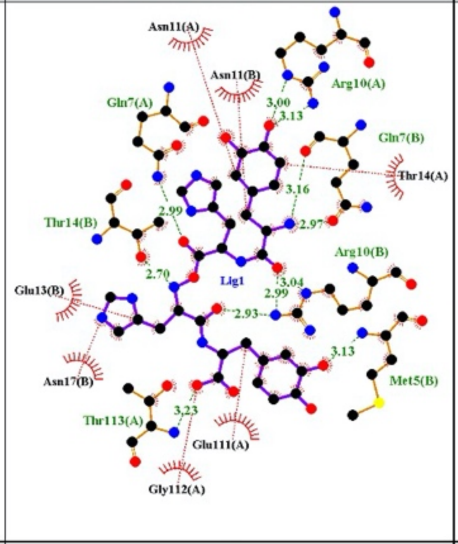	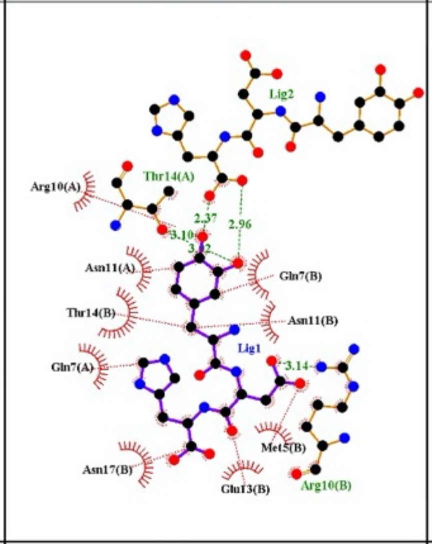	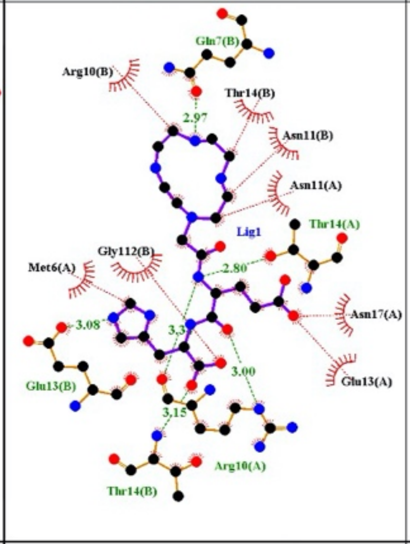	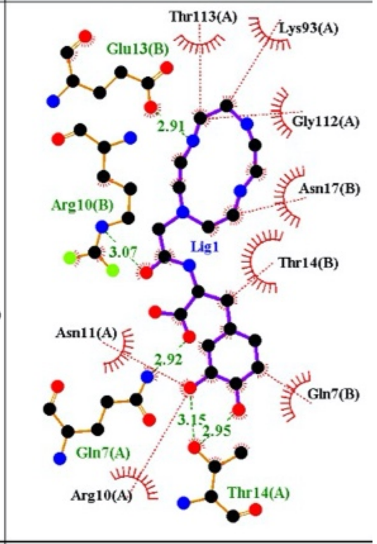
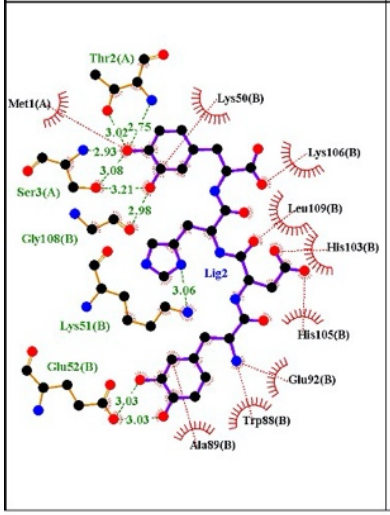	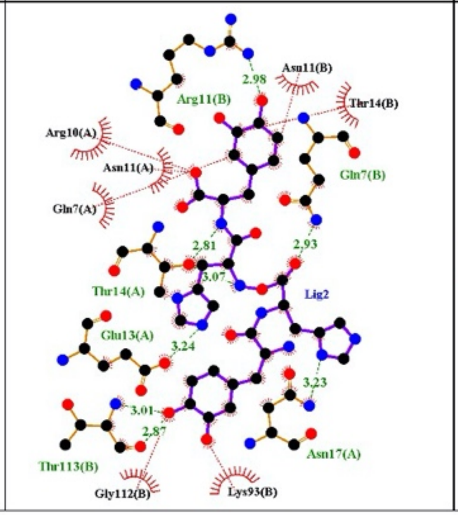	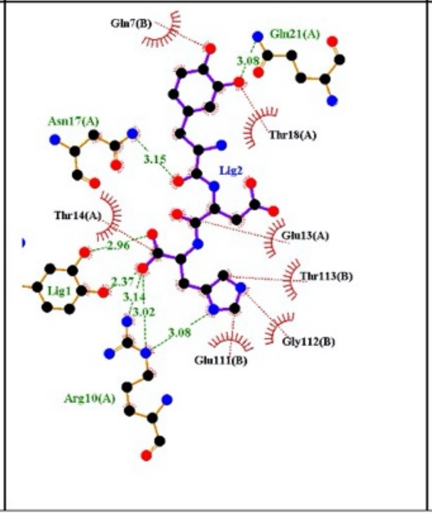	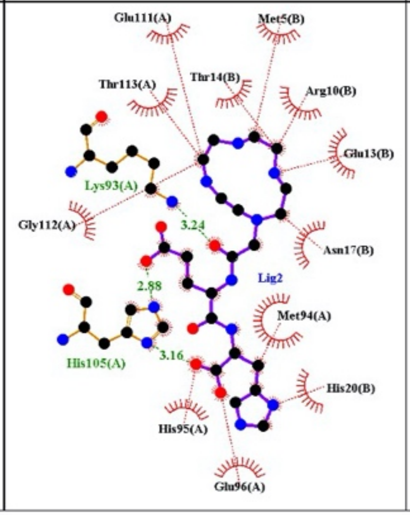	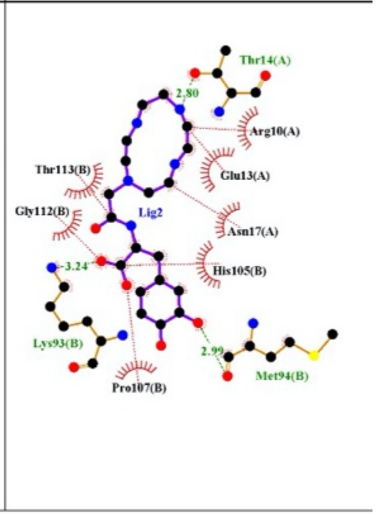

Figure S2. Binding sites in S100A9 homo-dimer for DOPA and Cyclen-based compounds captured in a representative all atom MD simulation time frame. LigPlot was used to map the binding sites for each ligand (http://pubmed.ncbi.nlm.nih.gov/7630882/). Ligands 1 and 2 are denoted in order of their mobility on protein surface as in Figure 6. The specific ligands are highlighted at the top of figure and are shown by ball-and stick models; carbon atoms are presented by black balls, oxygen – by red and nitrogen – by blue, respectively; ligand bonds are shown in purple. The side chains of S100A9 specific amino acids involved in ligand binding are also presented by ball-and-stick models with the same colour coding of atoms, in addition here sulphur atoms are shown by yellow balls; bonds are shown in brown; the names of amino acid residues and interaction distances are highlighted in green. The positions for the amino acids that form Van der Waals contacts with the ligands are marked by brown circular arches; they have potential to form H-bond interactions in different MD time frames (Figure 6) due to protein surface mobility.

DOPA-D-H-DOPA

Lig1 **Lig2**

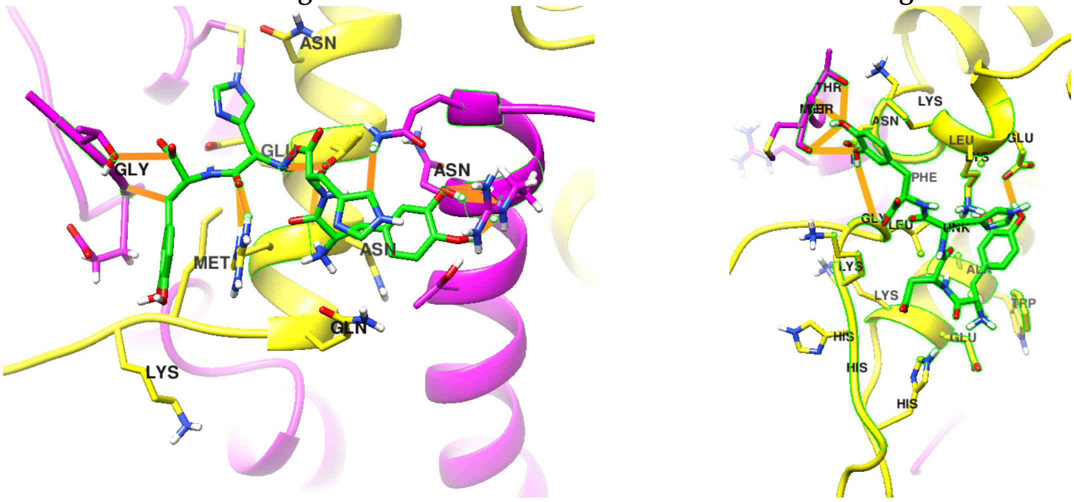

DOPA-H-H-DOPA

Lig1 **Lig2**

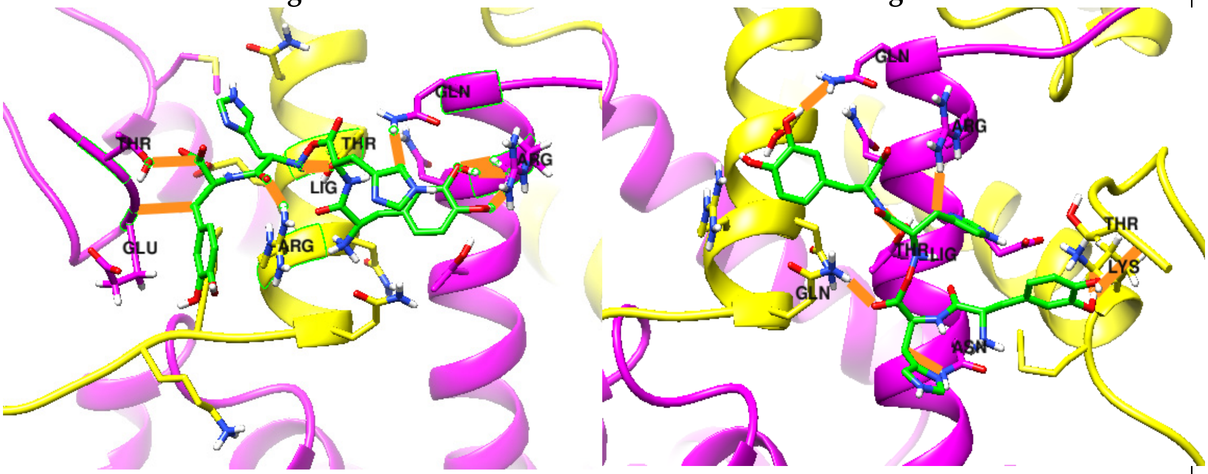

DOPA-D-H

Lig1 and Lig2

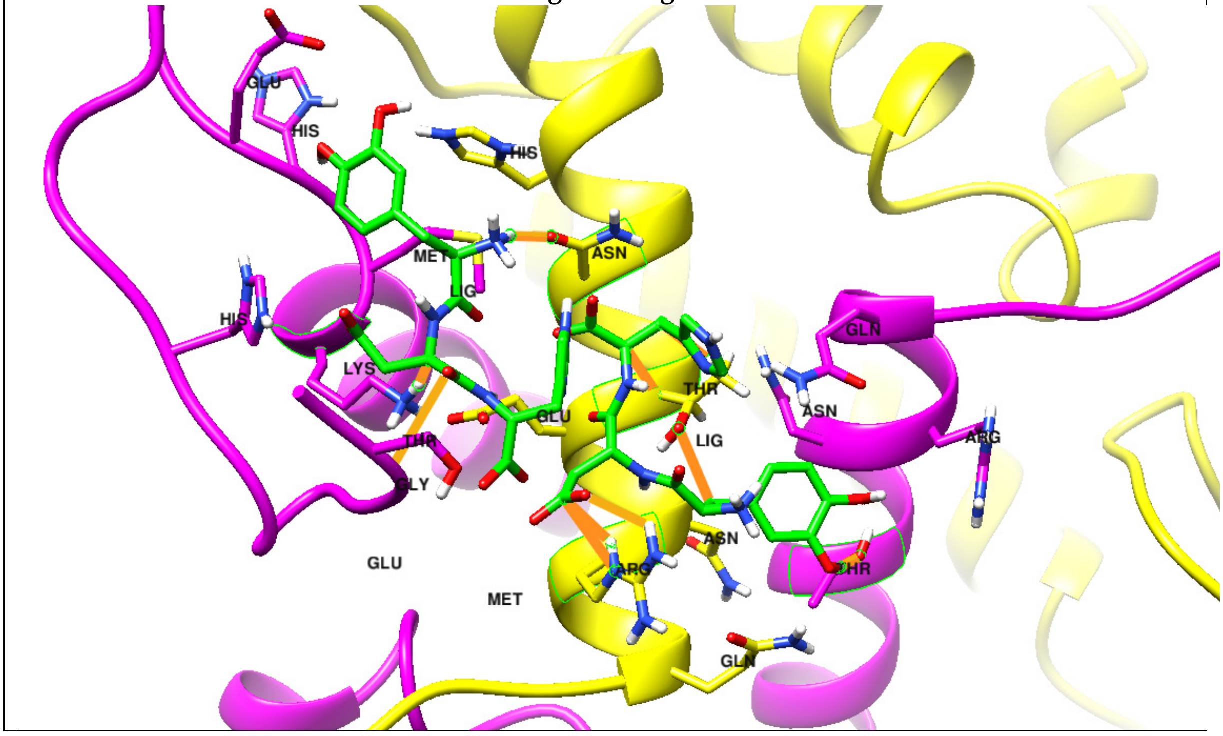

H-E-Cyclen

Lig1

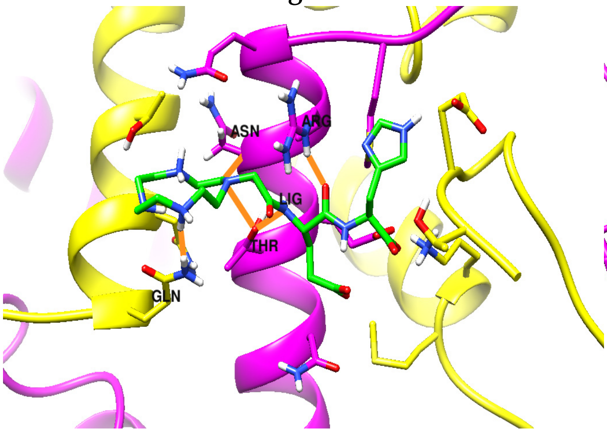

Lig2

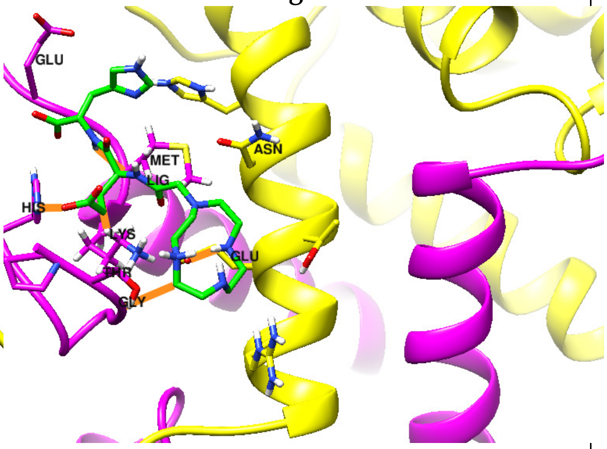

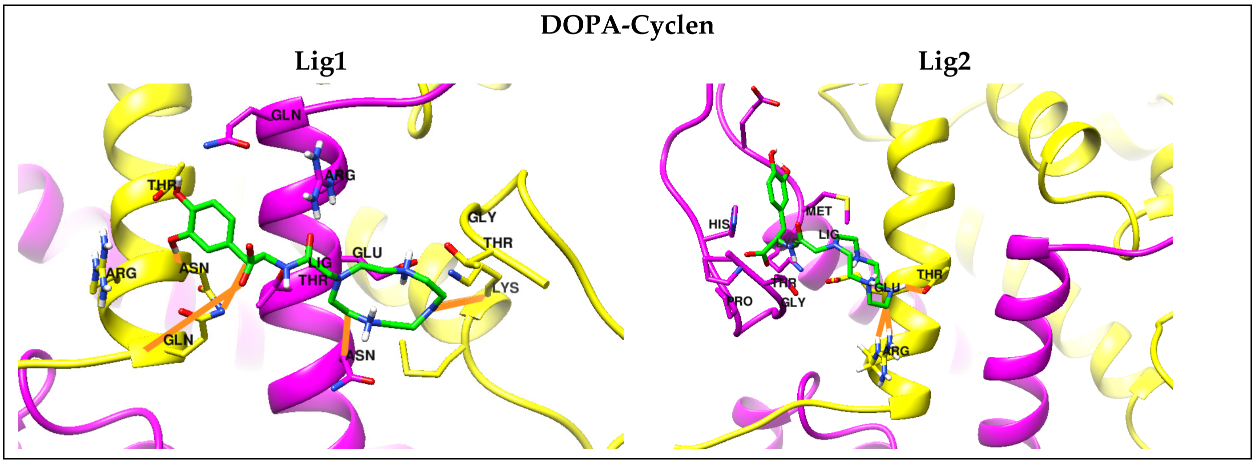

Figure S3. Zoomed panels of binding sites of DOPA and Cyclen-based compounds in S100A9 homodimer derived from MD simulation studies. The presented structures were derived from one of the MD steps to show the key residues involved in interaction at the atomic level. Ligands 1 and 2 are labelled in order of their mobility on protein surface as demonstrated in RMSD plots in Figure 6. The specific ligands are highlighted at the top of each figure. S100A9 monomers are depicted as yellow and magenta ribbons, respectively, as in Figure 5. The ligands are depicted by green sticks, where oxygen atoms are shown in red, nitrogen — in blue and hydrogen — in white, respectively. The names of S100A9 specific amino acids involved in ligand binding are marked. The orange line shows protein-ligand contacts for the selected MD frame. For DOPA-D-H complex Ligand 1 and Ligand 2 sites are shown together to indicate that the two ligands can interact when bound to each corresponding site.

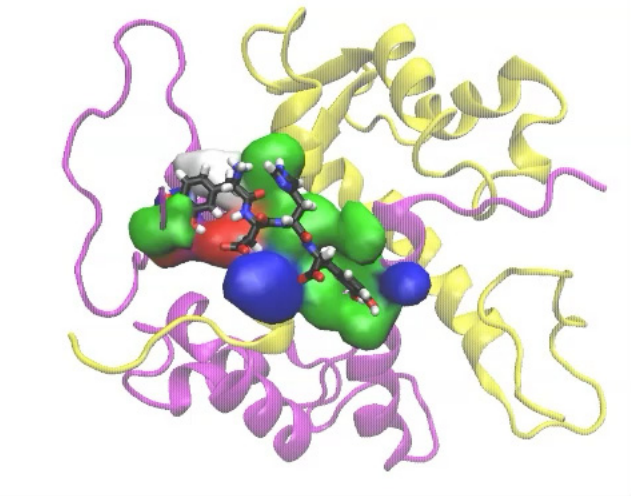

Video S4. Visual depiction of MD changes in S100A9 homodimer complex with DOPA-D-H-DOPA at the Ligand 1 site. The video gives a visual presentation of dynamic changes in protein-ligand complex during 100 ns MD simulation time as depicted also by RMSD and H-bond number changes in Figure 6. Ligand 1 is the most stable ligand staying on protein surface during all time of simulation. The video shows that polar ligand makes numerous interactions with the polar protein surface, which are very dynamic in nature due to ligand and protein surface mobility. The ligand is depicted by black sticks, where oxygen atoms are shown in red, nitrogen — in blue and hydrogen — in white, respectively. S100A9 monomers are depicted as yellow and magenta ribbons, respectively, as in Figure 5. The amino-acid side chains, which are within 4 Å distance from the ligand and form H-bonds or Van der Waals interactions with it, are shown by surfaces. Positive charged side chains are shown in blue, negatively charged – in red, polar and not charged–in green and hydrophobic –in white, respectively.

Video

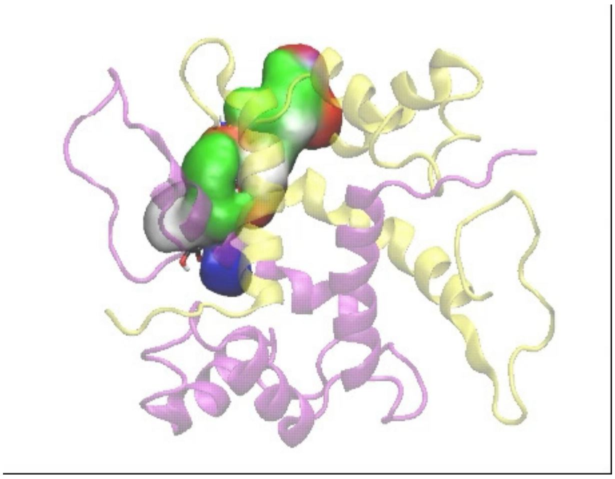

Video S5. Visual depiction of MD changes in S100A9 homodimer complex with DOPA-D-H-DOPA at the Ligand 2 site. The video gives a visual presentation of dynamic changes in protein-ligand complex during 100 ns MD simulation time as depicted also by RMSD and H-bond number changes in Figure 6. Ligand 2 is the next stable ligand, which stays on S100A9 surface only part time during MD simulation. The video shows that polar ligand makes numerous interactions with the polar protein surface, which are very dynamic in nature due to ligand and protein surface mobility. The ligand is depicted by black sticks, where oxygen atoms are shown in red, nitrogen — in blue and hydrogen — in white, respectively. S100A9 monomers are depicted as yellow and magenta ribbons, respectively, as in Figure 5. The amino-acid side chains, which are within 4 Å distance from the ligand and form H-bonds or Van der Waals interactions with it, are shown by surfaces. Positive charged side chains are shown in blue, negatively charged – in red, polar but not charged–in green and hydrophobic –in white, respectively.

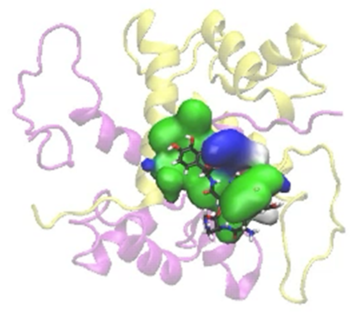

Video S6. Visual depiction of MD changes in S100A9 homodimer complex with DOPA-H-H-DOPA at the Ligand 1 site. The video gives a visual presentation of dynamic changes in protein-ligand complex during 100 ns MD simulation time as depicted also by RMSD and H-bond number changes in Figure 6. Ligand 1 is the most stable ligand remaining consistently on protein surface during all time of simulation. The video shows that polar ligand makes numerous interactions with the polar protein surface, which are very dynamic in nature due to ligand and protein surface mobility. The ligand is depicted by black sticks, where oxygen atoms are shown in red, nitrogen — in blue and hydrogen — in white, respectively. S100A9 monomers are depicted as yellow and magenta ribbons, respectively, as in Figure 5. The amino-acid side chains, which are within 4 Å distance from the ligand and form H-bonds or Van der Waals interactions with it, are shown by surfaces. Positive charged side chains are shown in blue, negatively charged – in red, polar but not charged–in green and hydrophobic –in white, respectively.

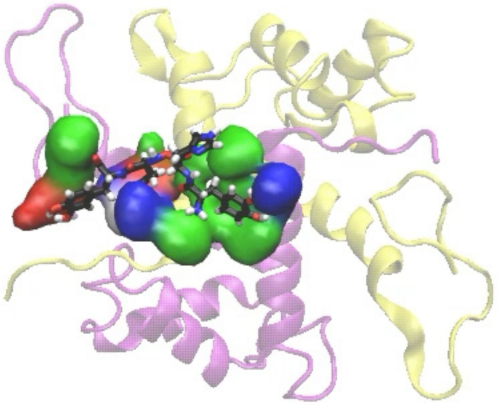

Video S7. Visual depiction of MD changes in S100A9 homodimer complex with DOPA-H-H-DOPA at the Ligand 2 site. The video gives a visual presentation of dynamic changes in protein-ligand complex during 100 ns MD simulation time as depicted also by RMSD and H-bond number changes in Figure 6. Ligand 2 is the next stable ligand, which also stays on S100A9 surface during MD simulation. The ligand is depicted by black sticks, where oxygen atoms are shown in red, nitrogen — in blue and hydrogen — in white, respectively. S100A9 monomers are depicted as yellow and magenta ribbons, respectively, as in Figure 5. The amino-acid side chains, which are within 4 Å distance from the ligand and form H-bonds or Van der Waals interactions with it, are shown by surfaces. Positive charged side chains are shown in blue, negatively charged – in red, polar but not charged–in green and hydrophobic –in white, respectively.
